# PSFHS: Intrapartum ultrasound image dataset for AI-based segmentation of pubic symphysis and fetal head

**DOI:** 10.1038/s41597-024-03266-4

**Published:** 2024-05-02

**Authors:** Gaowen Chen, Jieyun Bai, Zhanhong Ou, Yaosheng Lu, Huijin Wang

**Affiliations:** 1 Obstetrics and Gynecology Center, Zhujiang Hospital, Southern Medical University, Guangzhou, China; 2 Department of Electronic Engineering, College of Information Science and Technology, Jinan University, Guangzhou, China; 3 Auckland Bioengineering Institute, the University of Auckland, Auckland, New Zealand

**Keywords:** Physical examination, Medical imaging

## Abstract

During the process of labor, the intrapartum transperineal ultrasound examination serves as a valuable tool, allowing direct observation of the relative positional relationship between the pubic symphysis and fetal head (PSFH). Accurate assessment of fetal head descent and the prediction of the most suitable mode of delivery heavily rely on this relationship. However, achieving an objective and quantitative interpretation of the ultrasound images necessitates precise PSFH segmentation (PSFHS), a task that is both time-consuming and demanding. Integrating the potential of artificial intelligence (AI) in the field of medical ultrasound image segmentation, the development and evaluation of AI-based models rely significantly on access to comprehensive and meticulously annotated datasets. Unfortunately, publicly accessible datasets tailored for PSFHS are notably scarce. Bridging this critical gap, we introduce a PSFHS dataset comprising 1358 images, meticulously annotated at the pixel level. The annotation process adhered to standardized protocols and involved collaboration among medical experts. Remarkably, this dataset stands as the most expansive and comprehensive resource for PSFHS to date.

## Background & Summary

Detecting maternities at risk of requiring a cesarean section is paramount in enhancing perinatal outcomes and maternal satisfaction during childbirth. Prolonged labor or failure to progress is one of the common indications that causes approximately one-third of all cesarean deliveries, underscoring the vital need for precise prediction of prolonged labor to mitigate the occurrence of unplanned emergency cesarean procedures. Notably, the prevalence of cesarean section rates has witnessed a recent increase, often attributed to indications concerning the position of the fetal head (FH) and the progression of labor^1^.

Traditional methods involving subjective digital vaginal examinations for ascertaining FH position, rotation, and descent during delivery have demonstrated a lack of accuracy at times^2^. In this context, intrapartum transperineal ultrasound has emerged as an efficacious approach for monitoring FH descent. A critical advancement offered by this technique is the angle of progress (AOP), which serves as an objective, accurate, and reproducible indicator. Notably surpassing the limitations of digital vaginal examination^3^, the AOP offers insight into the relationship between the pubic symphysis (PS) and FH (PSFH). According to the practice guideline of the International Society of Ultrasound in Obstetrics and Gynecology (ISUOG), AOP is measured on a static 2D ultrasound image and is defined as the angle the angle between the long axis of the pubic bone and a line from the lowest edge of the pubic symphysis that tangentially touches the deepest bony part of the fetal skull. Research indicates that an AoP greater than or equal to 120 degrees is closely linked with a high chance of spontaneous vaginal delivery. Therefore, Therefore, AoP measured based on a single ultrasound image can be used as a predictive indicator of the mode of delivery.

The first step in interpreting the morphometrics is performing PSFH segmentation (PSFHS) - extracting visible PSFH contours from transperineal ultrasound images. However, PSFHS is a challenging task, involving accurate identification and delineation of the PSFH boundaries. FHs can vary widely in shape and orientation during different stages of labor, and surrounding structures like amniotic fluid and placenta can overlap with or obstruct parts of the head, introducing segmentation ambiguity. Additionally, the size and position of PSFH can vary significantly among individuals, making it difficult to develop a single generalized segmentation model. The inherent characteristics of ultrasound images, such as poor resolution, noise, and artifacts, further complicate the PSFHS process, especially during the dynamic changes in relative positions of PS and FH during the second stage of labor.

To address these challenges, automatic segmentation with Artificial Intelligence (AI) offers a promising approach^4^. Modifications to UNet structures have been introduced, incorporating attention mechanisms for improved detail capture^5,6^. Notably, Bai *et al*. devised a dual decoder strategy integrating traditional and deformable convolutions to concurrently extract morphological and global features^7,8^. Beyond architectural enhancements, Lu *et al*. explored the use of a shape-constrained loss function, reinforcing the UNet variant’s resilience to noise through the integration of a convex shape prior^9^. Despite the commendable performance of these UNet variations on proprietary datasets, their comparative evaluation remains challenging due to dataset diversity and size constraints. For example, Lu *et al*. released the JNU-IFM dataset^10^. Images of the JNU-IFM were acquired with the ObEye system from 51 pregnant women and collected from NanFang Hospital of Southern Medical University^10^. However, the development of AI models relies heavily on datasets from multiple centers, involving different patients, and sourced from various ultrasound devices. These factors significantly enhance the model’s performance, generalizability, and practical applicability in real-world clinical settings. Here are the key reasons why such diverse datasets are important: (1) Different centers may have patient populations with varying demographics, disease prevalence, and comorbidities; (2) Ultrasound devices from different manufacturers or even different models from the same manufacturer can produce images with varying qualities, resolutions, and characteristics; (3) Different centers might have varying protocols for image acquisition, patient preparation, and even image annotation standards; and (4) Images from different sources can have various types of noise, artifacts, or quality issues. A model trained on a diverse dataset is likely to generalize better to unseen data, reducing the risk of overfitting to specific characteristics of the training data. This is crucial for medical applications where the cost of errors can be very high. Therefore, larger and more comprehensive datasets are essential^8^.

To address these challenges and accelerate progress in AI research, it is imperative to promote data sharing and establish more comprehensive and representative datasets. In line with this objective, a proposed PSFHS dataset has been introduced, encompassing intrapartum transperineal ultrasound images that have been meticulously annotated at the pixel level through standard crowdsourcing among medical professionals. 1358 images from 1124 patients were gathered to form the PSFHS dataset. The accuracy of the pixelwise annotation, carried out by a group of trained students, was verified by expert physicians. This dataset is expected to facilitate the monitoring of labor progression through computer-aided systems and promote the practical implementation of technology in clinical settings, ultimately contributing to enhanced childbirth outcomes and improved care for both mother and fetus.

## Methods

### Subject characteristics

**Fig. 1:**
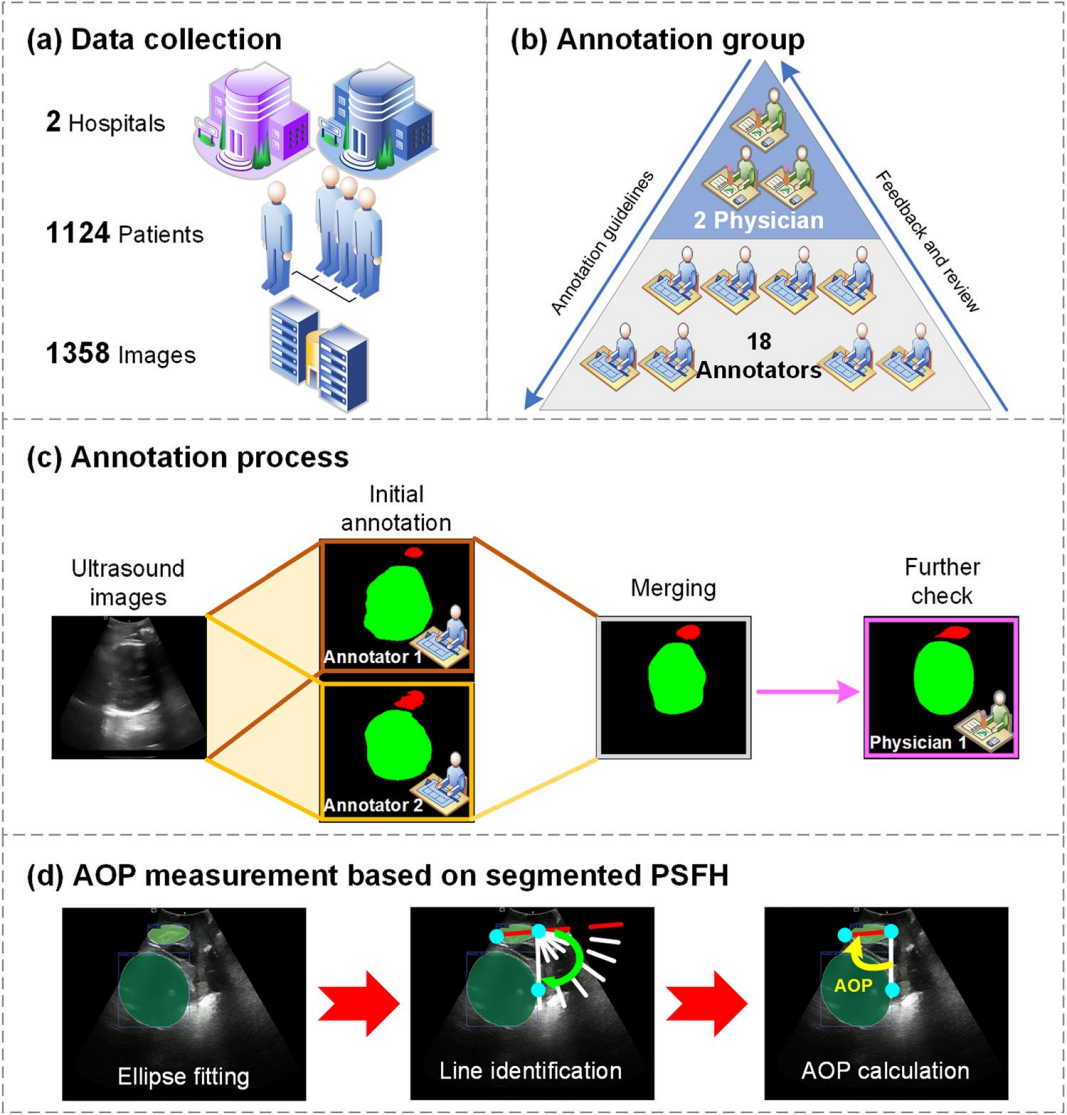
Workflow of the establishment of the proposed dataset. (**a**) 1358 images from 1124 pregnant women were collected. (**b**) The annotation team was made up of 2 physicians and 18 annotators. **(c)** For each ultrasound image, two annotators conducted initial annotation. These segmentations were merged and then adjusted by a physician to obtain the ground truth. (**d**) Based on the final ground truth of PSFH, AOP measurement consisted of ellipse fitting, line identification, and AOP calculation.

This retrospective image collection included 1124 pregnant women from two different medical institutions. Inclusion criterias were defined as: singleton pregnancy at term gestation (37 weeks or more), fetus in cephalic presentation, absence of documented fetal malformations. There are two parts in the PSFHS dataset: 1045 images from 1040 patients of Zhujiang Hospital of Southern Medical University, 313 images from 84 pregnant women from the Department of Obstetrics and Gynecology of the First Affiliated Hospital of Jinan University (Fig. 1a). This study received approval from the institutional review boards of Zhujiang Hospital of Southern Medical University (No. 2023-SYJS-023) and the First Affiliated hospital of Jinan University (No. JNUKY-2022-019). Informed consent was waived because of the retrospective nature of the study and the analysis used anonymous medical image data.

### Image acquisition

Ultrasound acquisitions were performed using a portable machine equipped with a 3.5 MHz probe. The ‘ObEye’ system (Guangzhou, China; http://lian-med.com) and the Esaote My Lab were used in the First Affiliated Hospital of Jinan University and Zhujiang Hospital of Southern Medical University, respectively. During each acquisition, the operator positioned the probe longitudinally in the translabial area to visualize both the PS horizontally in the upper central part of the image and the edges of the FH in the lower part^11^. These original images were cropped to remove sensitive information about patients.

### Image annotation

The team responsible for annotations included 2 proficient physicians and 18 students specializing in biomedical studies (refer to Fig. 1b). Before commencing their tasks, annotators received comprehensive training that involved familiarizing them with the structures of PSFH and the key aspects of ultrasound images. This training was facilitated through a combination of online sessions and in-person guidance by the physicians. Each annotator was assigned 15 test images, which were subsequently assessed by the physicians. If the annotations were deemed inadequate, the images were returned to the respective student for refinement. Annotators were instructed to utilize the pencil tool in Pair (https://www.aipair.com.cn/) for precise pixel-wise segmentation. They used red color for the pixel outlines of PS and green for the contour pixels of FH. In instances where the contours appeared fragmented or discontinuous, annotators were instructed to ensure that the contours maintained a complete elliptical shape. This instruction was essential considering the ultimate clinical application’s requirement to calculate AOP based on the segmented PSFH contours. The final segmentation ground truth was represented by a three-color image, where red pixels denoted PS, green pixels represented FH, and black pixels indicated the background. During the official annotation phase, each image was annotated by two annotators. Any overlapping pixels annotated by both annotators were further reviewed and adjusted by a highly experienced physician with a decade of expertise (refer to Fig. 1c). Note: Dropped artifacts in ultrasound images can significantly impact the quality and accuracy of the images, leading to potential errors in annotation. If artifact annotation may help to develop application-oriented robust algorithms, such as uncertain segmentation algorithms.

### Morphological parameters

Class imbalance is a common issue in image segmentation tasks that significantly affects the performance of deep learning models. When there is a class imbalance, it means that the number of pixels belonging to one class significantly outnumbers the pixels belonging to other classes. The pixel ratio of background to target (or among various targets) is a critical metric for understanding class imbalance. In the PSFHS dataset, the pixel proportions of PS, FH and background are, 1.78% ± 0.66%, 14.55 ± 5.73%, and 83.66 ± 6.27%, respectively. Based on the ground truth of PSFH, ellipse fitting is performed and thereby AOP is measured according to its definition—the angle between the longitudinal axis of the pubic symphysis and a line originating from its inferior edge to the leading edge of the fetal cranium tangentially (Fig. 1d)^7,9^. AOP is a predictor of the mode of delivery and the average value of AOP in the PSFHS dataset is 98.33° ± 21.11°.

## Data Records

All data records^12^ are available as files on the web page 10.5281/zenodo.10969427. The unzipped file folder of this dataset contains the original transperineal ultrasound images and annotation ground truth images. The unzipped file is organized into 2 folders, named “image_mha” and “label_mha”, that contain original transperineal ultrasound images and corresponding ground truth images, respectively. The images in these 2 folders are stored, named and arranged according to the same rule, where a specific image in the “label_mha” folder is the ground truth of the image with the same name in the “image_mha” folder. Images are named as “n.mha”, where “n” means the number of images. In the dataset, there are 1358 images (“n” from 03744 to 05101) of 1124 pregnant women. The images in the “image_mha” folder contain pixels labelled as 0, 1, or 2, where 0 represents the background, 1 represents the PS, and 2 represents the FH. These data can be accessed using the software “Insight Segmentation and Registration Toolkit”, available at https://itk.org/.

## Technical Validation

In this research, each ultrasound image underwent double annotation, followed by refinement by a medical professional. This process prompted the investigation of three distinct types of consistencies: intra-annotator consistency, referring to the same annotator at different time points; inter-annotator consistency among annotators at the same level; and inter-annotator consistency among annotators at different levels^13^.

To assess the intra-annotator consistency of the different annotators across various instances, a set of 40 images was selected from the complete dataset. These 40 images were annotated twice by three different annotators, including one physician and two others, on separate occasions. The Dice coefficient was then calculated between the annotations from the first and second rounds. The mean Dice coefficient for all 40 images and three annotators was 0.8817, with a confidence interval of 0.8502–0.9002.

To evaluate the inter-annotator consistency among annotators at the same level, discrepancies were assessed by computing the Dice coefficient between the annotations produced by the two annotators. The resulting mean Dice coefficient was 0.8750 (0.8520–0.8886).

Additionally, for the inter-annotator consistency among annotators at different levels, emphasis was placed on the first annotations. The Dice coefficient was used to measure the concordance between the physician’s mask and the annotations from the two annotators. The mean Dice coefficient for this scenario was determined to be 0.8720 (0.8520–0.8904).

Furthermore, to examine the intra-annotator consistency of the same annotator at different time points, the Dice coefficient was calculated between the first and second annotations. The resulting mean Dice coefficients were 0.8953 for the physician, 0.8811 for annotator 1, and 0.8689 for annotator 2.

Upon thorough analysis of both the original annotations and the illustrative set, it was concluded that the annotations demonstrated stability and consistency not only within a single annotator over different instances but also across various annotators. These findings collectively provide a strong basis for accurate annotation and reproducible PSFHS, as detailed in Table 1.

**Table 1:** Dice scores on an example set of 40 images.

	Physician	Annotator 1	Annotator 2
1st	89.04 ± 0.07	87.36 ± 0.05	85.20 ± 0.17
2nd	90.02 ± 0.12	88.86 ± 0.05	88.58 ± 0.12

## Usage Notes

The whole dataset used for the PSFHS challenge of MICCAI2023 (https://ps-fh-aop-2023.grand-challenge.org/) includes two parts^12,14,15^: one is this PSFHS dataset (10.5281/zenodo.10969427)^12^ and another is from the JNU-IFM dataset (10.6084/m9.figshare.14371652)^16^. These images in the PSFHS dataset can also be used for the Intrapartum Ultrasound Grand Challenge (IUGC) 2024 of MICCAI 2024 (https://codalab.lisn.upsaclay.fr/competitions/18413).

## Data Availability

No novel code was used in the construction of the PSFHS dataset.
